# The Role of Type I Interferons in the Pathogenesis and Treatment of COVID-19

**DOI:** 10.3389/fimmu.2020.595739

**Published:** 2020-09-30

**Authors:** Gideon Schreiber

**Affiliations:** Department of Biomolecular Sciences, Weizmann Institute of Science, Rehovot, Israel

**Keywords:** type I interferon, COVID-19, signaling, differential activity, inflammation

## Abstract

Type I interferons (IFN-I) were first discovered over 60 years ago in a classical experiment by Isaacs and Lindenman, who showed that IFN-Is possess antiviral activity. Later, it became one of the first approved protein drugs using heterologous protein expression systems, which allowed its large-scale production. It has been approved, and widely used in a pleiotropy of diseases, including multiple-sclerosis, hepatitis B and C, and some forms of cancer. Preliminary clinical data has supported its effectiveness against potential pandemic pathogens such as Ebola and SARS. Still, more efficient and specific drugs have taken its place in treating such diseases. The COVID-19 global pandemic has again lifted the status of IFN-Is to become one of the more promising drug candidates, with initial clinical trials showing promising results in reducing the severity and duration of the disease. Although SARS-CoV-2 inhibits the production of IFNβ and thus obstructs the innate immune response to this virus, it is sensitive to the antiviral activity of externally administrated IFN-Is. In this review I discuss the diverse modes of biological actions of IFN-Is and how these are related to biophysical parameters of IFN-I–receptor interaction and cell-type specificity in light of the large variety of binding affinities of the different IFN-I subtypes towards the common interferon receptor. Furthermore, I discuss how these may guide the optimized use IFN-Is in combatting COVID-19.

## Introduction

**Figure 1:**
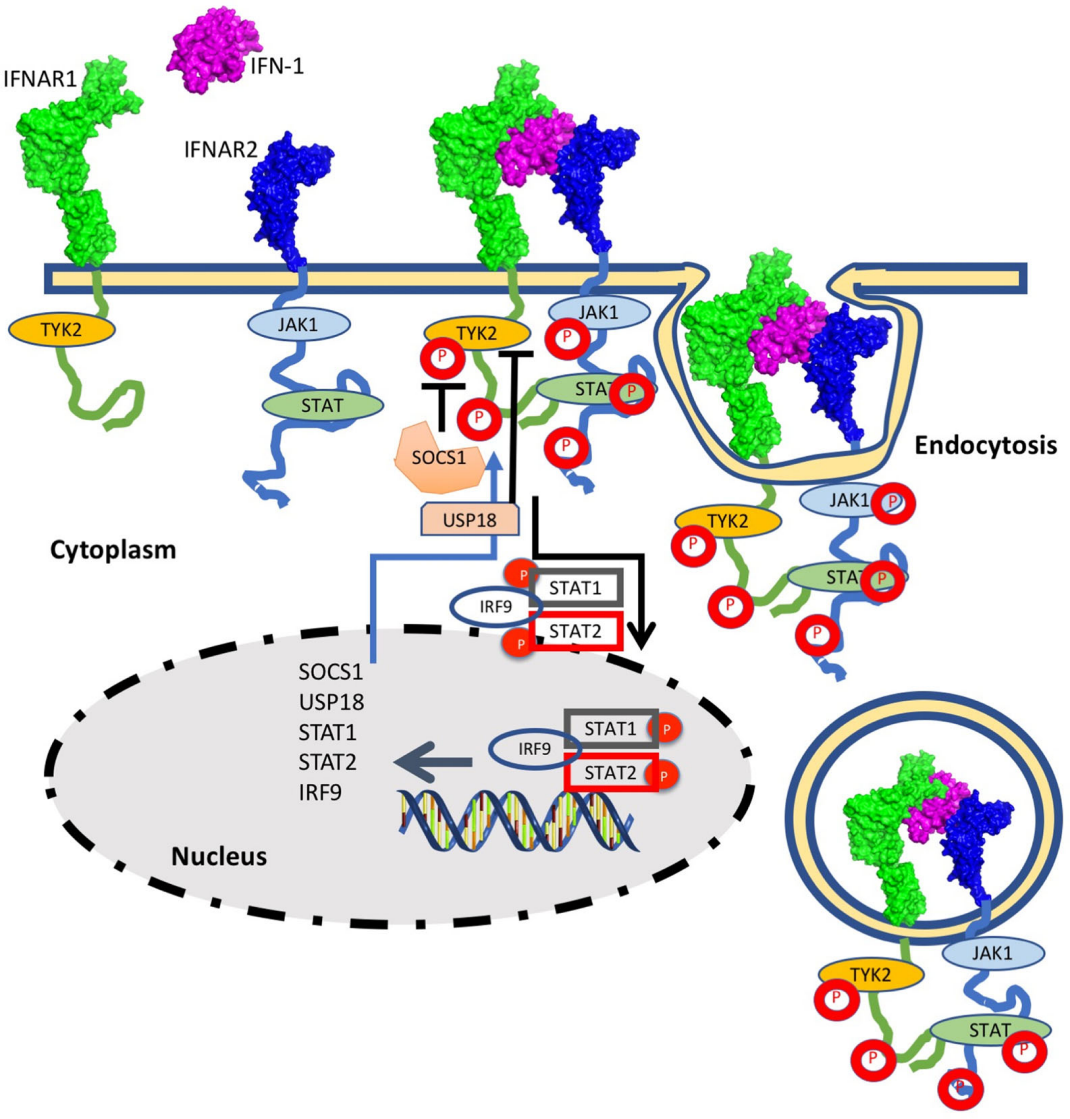
The interferon response is initiated by IFN-I binding to the extracellular domains of IFNAR1 and IFNAR2. Following ternary-complex formation, the associated JAK kinases cross-phosphorylate each other as well as the associated STATs and tyrosine residues on the intracellular domains of the receptors. Upon phosphorylation the STATs are released and are transported to the nucleus. The STAT1/STAT2/IRF9 complex is strongest associated with IFN-I induced gene induction, albeit other STAT complexes are activated as well (see **Figure 2** for details). The STAT complexes serve as transcription factors for many IFN-I induced genes. Three main feedback mechanisms quell IFN-I activity: Receptor Ubiquitination, resulting in receptor endocytosis (which is initiated within minutes from IFN-I induction) and SOCS and USP18, which are IFN-I induced genes and thus their feedback relates to their production to high levels (which takes hours).

**Figure 2:**
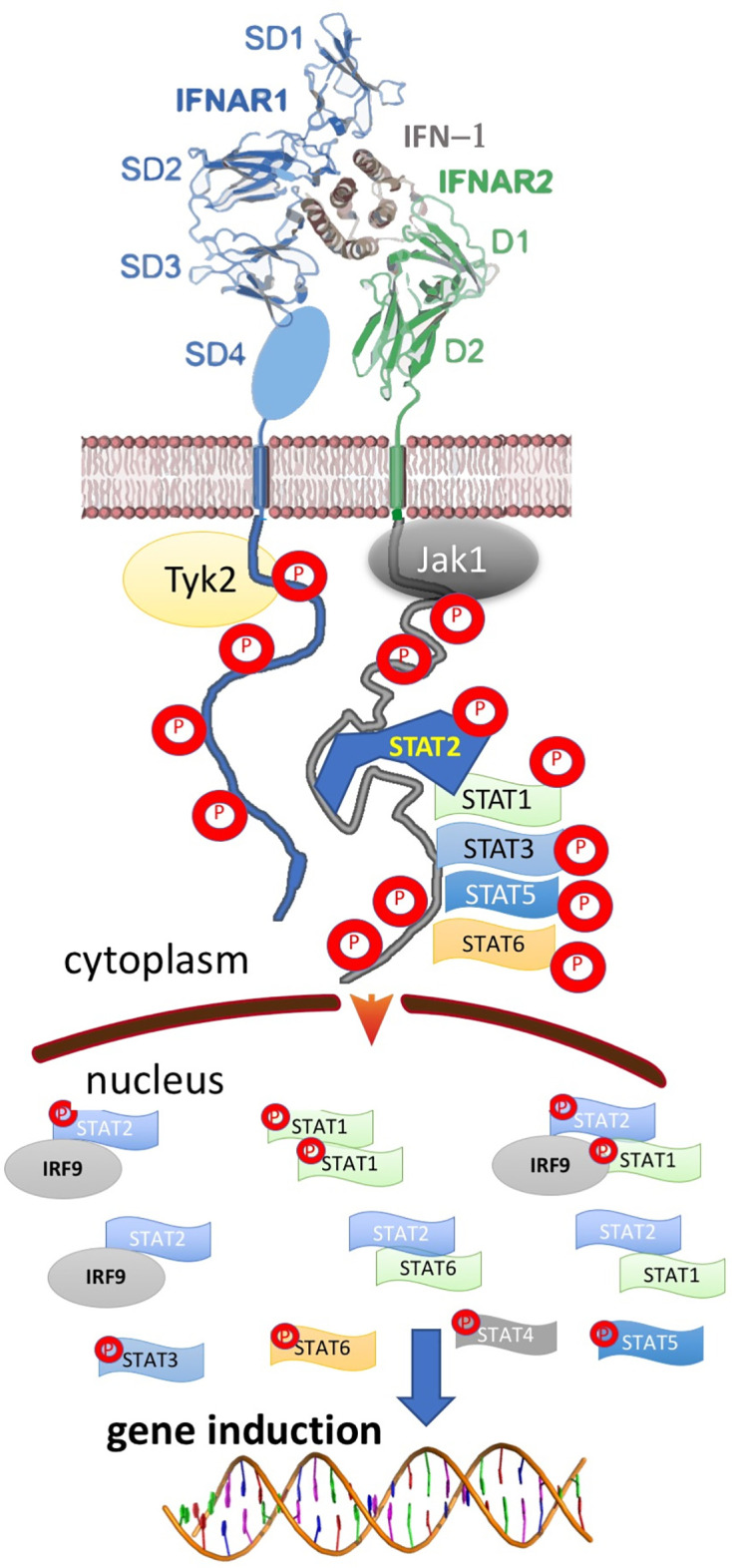
Ternary, IFN-I/IFNAR1/IFNAR2 complex formation results in the activation of multiple STAT complexes that serve as transcription factors for different genes. The activated STATs and IFN-I regulated genes vary between different cells, IFN-I subtype, its concentration and duration of activation, result in a pleiotropy of responses.

Type I interferons (IFN-I) are a family of cytokines that bind the type I interferon receptor, constituted of two transmembrane subunits, IFNAR1 and IFNAR2 (**Figure 1**). The two receptors are constituted of an extracellular domain, which binds IFN-I, a transmembrane helix and an unstructured intracellular domain (ICD) that binds JAKs and STATs (1, 2). JAK1 is associated with IFNAR2 and TYK2 with IFNAR1. STAT1 and STAT2 (and maybe also other STATs) were found to be constitutively bound to the ICD of IFNAR2 (3–5). Binding results in close proximity of the intracellularly associated JAKs, JAK1 and TYK2, resulting in their activation through cross phosphorylation (**Figure 1**) (6, 7). This also results in receptor phosphorylation, which role is still under debate (3, 8–10). The phosphorylated STATs dissociate from the receptor and form homo and hetero dimers, which are transported to the nucleus, where they serve as transcription factors for a large number of genes. The most prominent effects are associated with STAT1/STAT2 heterodimerization, which together with IRF9 form the interferon-stimulated gene factor 3 (ISGF3), which bind a distinct group of target genes harboring the interferon-stimulated response elements (ISRE). In addition to this, IFN-I drives STAT1/STAT1 and STAT3/STAT3 homodimerization, the formation of a STAT2/IRF9 binary complex and more (6, 10–12) (**Figure 2**). This leads to the transcription activation or suppression of over 1,000 genes, which drive a wide range of innate and adaptive immune functions. These, in turn respond against various pathogens, act as important regulators in tumor immunity and have a role in pathophysiology and autoimmune diseases (10, 13–18). STAT2 knockout cells still activate a STAT1/STAT1 response mediated by IRF1, while STAT1 knockout cells activate a STAT2/IRF9-induced response (10). Surprisingly, no change in the gene induction relative to wild-type cells was observed in STAT3 knockout HeLa cells, despite the strong IFN-I–induced phosphorylation of STAT3. However, as IFN-I responses are cell-type specific, a STAT3/STAT3-induced response may still be found in other cells than HeLa.

Due to this wide range of physiological responses, IFN-I has provided therapeutic benefits for multiple diseases, including multiple sclerosis, some cancers and viral diseases (hepatitis B and C) (19–21). Due to the efficient activation of antiviral activities by IFN-Is, most viruses have contemplated mechanisms to avoid its actions (22–24). For example, the Ebola virus, which outbreak in central Africa killed tens of thousands of people (25, 26), avoids IFN-I activity by producing the VP24 protein that binds the karyopherin alpha nuclear transporter. Thereby, it inhibits the nuclear transport of phosphorylated STAT1, rendering cells refractory to IFN-Is.

Another example of viral mechanisms that evolved to eliminate IFN-I functions in inducing innate immunity is given by the SARS corona virus, where both the production of IFNβ and the IFN-I induced signaling are attenuated. Recently, a more infective version of SARS has emerged, SARS-CoV-2 (which causes the COVID-19 disease). COVID-19 cases have been first reported by the end of 2019 in China, and rapidly became a world-wide epidemic with unprecedented consequences (27, 28). SARS-CoV-2 seems to have originated from horseshoe bats. Similar virus strains that circulate in bats in Hubei province in China may in the future cause further new zoonotic outbreaks (29). SARS-CoV-2 has 83% homology to the SARS-CoV virus that also spread from China in 2002 (30). SARS-CoV-2 proved to be much more infectious compared to the original SARS virus, resulting in a global epidemic. As IFN-I drives strong antiviral activities, the mechanisms SARS-CoV and SARS-CoV-2 combat IFN-I activities has been a matter of intense research, with at least 6 proteins being identified to counteract IFN-I functions in the SARS-CoV virus (31). In addition, IFN-Is were implicated in contributing to the severity of the cytokine storm, which is a major complication of SARS-CoV and SARS-CoV-2 and can lead to respiratory distress syndrome (ARDS) and death (31, 32).

In this review I will describe our current knowledge on the involvement of IFN-Is in the development of the COVID-19 disease, and how this relates to the different activities associated with type I interferons.

## Common and Unique Features of Type I Interferon Signaling

Type I interferon receptors are found on all cell types, and are a major component of the innate immune system. Human type I interferons include 13 similar IFNαs with 80% homology between them and single IFNω, κ, ϵ and β, with lower homology (30–50%). All of them bind the receptor complex, composed of IFNAR1 and IFNAR2 at the same proximal location (1, 2, 33). Despite structural similarities among the ternary IFN-I-IFNAR1-IFNAR2 complexes, IFN-Is drive a range of different activities, dependent on the cell type and the interferon subtype (34). This apparent paradox has major implications for understanding the role of IFN-I in health and disease and its varied applications as a drug against a pleiotropy of diseases.

IFN-I signaling is initiated by binding of IFN-I to its receptor. It has been suggested that cytokine receptors are pre-associated, with ligand binding activating signaling through the induction of conformational changes (35). However, more recent single-molecule receptor tracking on life cells has clearly shown that for many of the cytokines, its role is to bring the receptors into close proximity, which drives signaling (36). This seems to be the case also for IFN-I induction, as shown both using single receptor tracking and mutational analysis (**Figure 1**) (37, 38). While structurally, the ternary ligand-receptor complex seems to be the same for all IFN-Is, the binding affinity differs by many orders of magnitude. The tightest binding IFN-I is IFNβ, which binds IFNAR1 with 100 nM affinity and IFNAR2 with sub-nanomolar affinity. The different IFNα subtypes bind IFNAR1 with 0.5 to 5 µM affinity and IFNAR2 with 1 to 100 nM affinity, with IFNα1 being the weakest binding IFNα (39, 40). Even weaker binding was measured for IFNϵ, with ~100-fold reduced affinity relative to IFNα proteins (15). Interestingly, IFNϵ is constitutively expressed by the reproductive tract epithelium and is regulated by hormones during the estrus cycle, reproduction, menopause and by exogenous hormones. Thus, its mode of action is different from other IFN-Is (41).

These large differences in binding affinity between IFN-I subtypes were suggested to result in major differences in biological activity. To obtain a better insight into the molecular mechanisms of their actions, IFNα2 was engineered to cover the whole range of binding affinities of natural IFN-Is to both the high affinity (IFNAR2) and low affinity (IFNAR1) receptor chains (1). These studies have shown that indeed, the binding affinity to both receptors is a major determinant of IFN-I activity (42). Using both natural and engineered IFN-Is has shown that even weak binding IFN-Is activate the cellular antiviral program at very low (pM) concentrations (39). Moreover, the antiviral program was activated in all cell-lines tested. Despite the 50-fold higher affinity of IFNβ over IFNα2 towards binding IFNAR receptors, its potency to elicit an antiviral response is similar. For example, in WISH cells (originally thought to be of amniotic origin, but later found to be a HeLa (cervix cancer) contaminant) the EC_50_ for antiviral activity of IFNα2 is 0.3 pM, while the EC_50_ for IFNβ is 0.15 pM (43). WISH cells have been extensively used to characterize IFN-I activity, including for definition of IFN-I unit activity. An upper limit for antiviral potency was further verified by engineering an IFNα2 variant, YNS-α8-tail, with 50-fold tighter binding to IFNAR1 and 15-fold tighter binding to IFNAR2 in comparison to IFNα2 (thereby surpassing the receptor binding affinity of natural IFNβ). Still, the EC_50_ for antiviral activity is only 3-fold lower in comparison to IFNα2 (44, 45).

Conversely to antiviral activity, IFNβ is much more potent in activating the antiproliferative program relative to IFNα2, a result that was also verified using the IFNα2 variant, YNS-α8-tail (45). The EC_50_ for antiproliferative activity on WISH cells is 2 nM for IFNα2, 50 pM for IFNβ and 20 pM for YNS-α8-tail. A similar increase in antiproliferative potency was observed also for OVCAR3 and HeLa cells. Interestingly, while antiviral activity was observed in all cell lines tested, some cell lines were not susceptible to IFN-I induced antiproliferative activity (for example T47D and K562), independent on the concentration and subtype of IFN-I (45).

**Figure 3:**
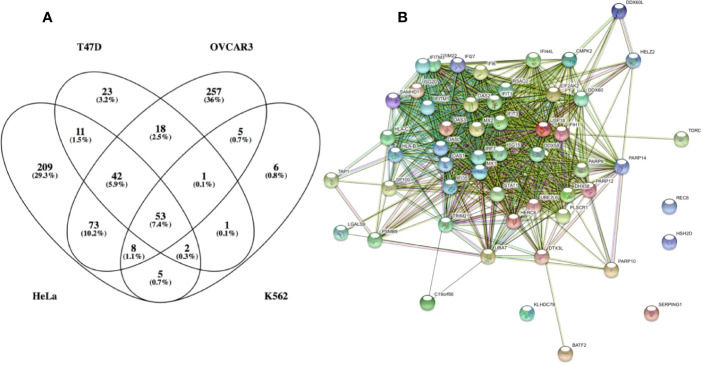
Genes which expression was upregulated by over 3-fold in the following cell lines: HeLa, T47D, K562 and OVCAR3. **(A)** venn diagram of the upregulated genes. **(B)** STRING: functional protein association network analysis of upregulated genes in all 4 cell lines (53 genes). According to STRING and GO analysis, the commonly upregulated genes have a strong antiviral signature. The top GO terms (FDR <10^−25^) are response to type I interferon, innate immune response, response to virus, defense response and immune system process. It is interesting to note that antiviral genes constitute most of the upregulated genes common to all 4 cell lines. Antiviral genes are also the majority of upregulated genes in K562 and T47D cells. Conversely, OVCAR3 and HeLa cells have many unique upregulated genes, many of them related to immunomodulatory functions, cell cycle, apoptosys and more.

To better understand the molecular basis for this finding, IFN-I induced gene expression was monitored using various IFN-I subtypes or engineered mutants on the background of different cell-lines. These experiments showed that low concentrations of weaker binding interferons activate the expression of mostly antiviral genes. Higher concentrations of interferons activate also other genes, many of them related to immune-modulation (45). Examples for such genes are chemokines such as CXCL10 and 11, which are involved in chemotaxis of T cells and natural killer cells, induction of apoptosis, regulation of cell growth and more. We gave the term of “robust” for the common IFN-I induced program (including its antiviral activity) and “tunable” for the other programs induced by IFN-Is, which include between others antiproliferative and immunomodulatory activities (34). Further investigations into these two programs has shown that cells with low receptor numbers activate only the robust program, and that not all cell types execute the tunable program, conversely to the robust program that is common to all cells (46). Tighter binding IFN-Is at higher concentrations are essential for the activation of the tunable program. Genes upregulated by the robust program are mostly classical antiviral genes, such as MX1 and MX2, OAS1 and 2, PKR, IFIT1, 2 and 3, ISG15, and many more. **Figure 3A** shows a Venn diagram of RNAseq data for 4 different cell-lines induced with IFN-I. The diagram shows that 53 genes are commonly upregulated by all 4 cell-lines. **Figure 3B** shows STRING protein interaction analysis of these common genes. Clearly, these form a tightly interacting mesh of gene products. Gene Ontology analysis shows these genes to have an extremely high signature for antiviral activity and IFN-I activation. Promoter analysis of common ISGs has shown them to be driven by the classical ISRE promoter sequence (45). Conversely, for tunable genes no clear promoter sequence was identified. The exact mechanism of how tunable genes are upregulated by IFN-I is thus not yet fully understood.

## Interferon and Inflammation

From an immunological point of view, IFN-Is have three major functions: 1. To activate an antiviral state in infected and neighboring cells that limits spread of infection. 2. Modulate innate immune responses, including antigen presentation and natural killer cell functions while restraining pro-inflammatory pathways. 3. activating the adaptive immune system for the development of high-affinity antigen-specific T and B cell responses (47). As IFN-Is are highly active molecules, their expression and signaling potency is highly regulated. Opposing augmenting and suppressive signals are induced by host factors. Suppressive pathways include IFN-I activation of USP18, an ISG that suppresses signal transduction by reducing the ability of IFN-Is to form an active receptor complex (38, 48). A second inhibitory mechanism is the induction of SOCS1 and SOCS3, which KIR domain block the substrate binding groove on JAK, thereby inhibiting STAT phosphorylation (49). A third mechanism is by rapid endocytosis and subsequent lysosomal degradation of activated IFNAR complexes (50, 51) resulting in reduced receptor numbers (**Figure 1**). It has been demonstrated that a mutant in IFNAR1 (S535A and S526A in human and mouse respectively), which fails in IFNAR1 endocytosis through blocking its ubiquitination result in high incidence of inflammation (51, 52). At the transcriptional level, IFN-I response can also be regulated by miR-155, which is highly induced by pattern recognition receptors and inflammatory signaling, and suppresses the expression of over 100 genes. Between them genes related to the interferon pathway. It was shown that miR-155-deficient CD8(+) T cells had enhanced type I interferon signaling and were more susceptible to interferon’s antiproliferative effect (53).

High basal IFN-I levels are implicated in various immunological diseases, such as systemic lupus erythematosus and more (18, 54, 55). However, IFN-I has also anti-inflammatory effects, as best demonstrated by their ability to suppress multiple-sclerosis (56). It is important to note that beneficial results in treating multiple-sclerosis were observed only for IFNβ but not for IFNα treatment (56). To see whether this relates to the higher receptor binding affinity of IFNβ, we established a transgenic mouse harboring the human interferon-receptors extracellular domains fussed to the mouse intracellular domains and compared the severity of EAE in a mice model upon treatment with IFNα2, IFNβ and the high-affinity engineered IFN-YNS-α8-tail. We found that the IFN-YNS-α8-tail had the strongest suppressive effect on the development of EAE (57). The effect was further enhanced by PASylation of IFN-YNS-α8-tail, which extends it plasma half-life by 10-fold. Interestingly, we found a tight relation between the increased levels of expression of PD-L1 in mice and the severity of the disease. These data show that tight binding IFN-Is induce preferential anti-inflammatory responses, at least in this MS mouse model. Another example for the immunosuppressive activity of IFN-I was shown for LCMV infection, which induces consistent IFN-I production including the immunosuppressive factors IL-10 and PD-L1 (58). In addition to the above, Interferons contribute to inflammasome activation through several different mechanisms, including caspase-11 expression and the IFN-I inducible GBP protein expression, which was reported to have an important role in caspase-11 activation and pyroptotic cell death (59).

IFN-Is have important roles in protecting the lung from spread of respiratory viruses. In addition to their direct role, IFN-Is have also been found to be critical in initiating lung inflammatory responses, by inducing recruitment and activation of immune responses, which have to be kept under control. IFN-Is have been shown to result in the production of chemokines such as CCL2 and CXCL10, which play important roles in the recruitment of monocytes/macrophages, T cells, NK cells, and DCs, therefore directly influencing inflammation in the lung (60). This varied effect of type I IFNs on T cells is partly dependent on the different STATs induced by type I IFNs. In the absence of IFN-Is, the detection of accumulating viral RNA and downstream processing of the signal is compromised, leading to viral spread and also to reduced inflammation in the lung. Interestingly, there is an age-related reduction of IFN-I production and ISG induction after viral infection, which may be related to the higher susceptibility of elderly population to lung infections (61).

## A Constant Battle Between the Interferon System and Viruses

Viruses have developed many strategies to interfere with the synthesis of IFN-Is or the IFN-I induced responses. One of them, is the stimulation of turnover of the interferon receptors. Among other viruses implicated in accelerating the turnover of IFNAR1 are EBV, herpes simplex virus, hepatitis C and B viruses, vesicular stomatitis virus and the SARS coronavirus (62, 63). SARS-CoV has been shown to suppress IFN-I responses in the host through multiple mechanisms. A subdued IFN-I response diminishes antigen presentation and reduces the antiviral adaptive Th-1 immune response. IFN-Is communicate between cells against pathogens and have a critical role in the immune system, such as activating natural killer (NK) cells and macrophages. In addition, IFN-Is cause flu-like symptoms, which are observed in various diseases. These symptoms may have a role in alerting a person of his/her sickness, in order to limit disease-spread to other individuals. In SARS-CoV and MERS-CoV, the induction of IFNβ is suppressed altogether. This dampening approach is highly associated with the disease severity and increased mortality (64). In the lethal cases of SARS-CoV or MERS-CoV infections, the increased influx of inflammatory cells is always observed. In a mouse model of SARS- CoV infection, imbalance in IFN-I and inflammatory cells were shown as the main cause of fatal pneumonia (65). In addition to these, SARS-CoV implements strategies to evade the immune response by antagonizing IFN-I induced signaling pathways. The ORF6 protein blocks the expression of STAT1-activated genes (66). SARS-CoV and MERS-CoV encode papain-like protease (PLP) that is able to impede the immune response function (67). In addition, SARS-CoV interacts with ISG15 and antagonizes the IFN-I-mediated antiviral response (68). The MERS-CoV ORF4b antagonizes the antiviral IFNβ production by inhibiting IRF3 and IRF7 (69). Also SARS-CoV inhibits activation of IRF3/7, slowing IFNβ production upon infection (70). While IRF3 is expressed in many different cell types, plasmacytoid dendritic cells are the only cells constitutively expressing IRF7 (47).

IFN-I treatment has been studied against MERS-CoV and SARS- CoV in numerous experiments, both *in vitro* and *in vivo*, and in combination or not with lopinavir/ritonavir, ribavirin, remdesivir, corticosteroids, or IFNγ. While IFNα and β were efficient *in vitro* and in certain animal models, their success in humans was less convincing [for review see, (71, 72)]. It should be noted that reduction in ARDS mortality (not related to SARS) was also found to be at best marginal upon treatment with IFN-I (73). Still, one has to consider that mice studies have shown the timing of IFN-I administration to be critical, with positive effects being observed if IFN-I was administered shortly after infection. Conversely, IFN-I failed to inhibit viral replication and resulted in unwanted side-effects when administered later in the disease circle (74, 75). These include elevated lung cytokine/chemokine levels, vascular leakage, and impaired virus-specific T cell responses. It is interesting to note that a knockout of the IFN-I receptor in mice resulted in its protection from lethal SARS-CoV infection. These findings have major implications on how to treat humans against SARS and MERS, and could have affected the outcome of the clinical studies.

### Mode of Infection by SARS-CoV-2

The COVID-19 pandemic started in December 2019 in Wuhan, China. By the summer of 2020, thirty million cases were reported worldwide, with over 900,000 fatalities. As COVID-19 is closely related to the SARS-CoV virus, the interest in the effect of interferons on its disease progression, and its potential as a drug was immediate. Disease progression of COVID-19 goes through a number of stages. The initial stage, which last from 2 to 14 days (usually 5–6 days) from infection is asymptomatic. A certain proportion of patients never produce any symptoms (the percentage of those is under debate, but a range of 30–50% is most likely). Of those who develop symptoms, they are mostly mild (80% of those who develop symptoms). From the remaining 20%, about half will develop severe symptoms, which require hospitalization in intensive care units. The mortality rate, from those developing symptoms is 2% to 5%. The numbers given above are average, and change dramatically with age. At young age most of the infected people will be asymptomatic, while over the age of 70 about 80% will have symptoms. Moreover, as the age progresses, symptom severity increases (76). The major complication of severe infection is pneumonia, which can develop into acute respiratory distress syndrome (ARDS). In addition, COVID-19 has been linked to cardiovascular sequelae, such as myocardial injury, arrhythmias, cardiomyopathy and heart failure, acute kidney injury, neurological complications, and acute ischemic stroke (28). Developing severe symptoms and death is strongly related to background conditions. The strongest relation is to age, with the risk to people under 50 being very small, while the risk peaks for people over the age of 75. In addition, chronic kidney disease, chronic obstructive pulmonary disease, immunocompromised state, obesity, heart conditions and type 2 diabetes are linked to higher incidents of sever disease (76).

**Figure 4:**
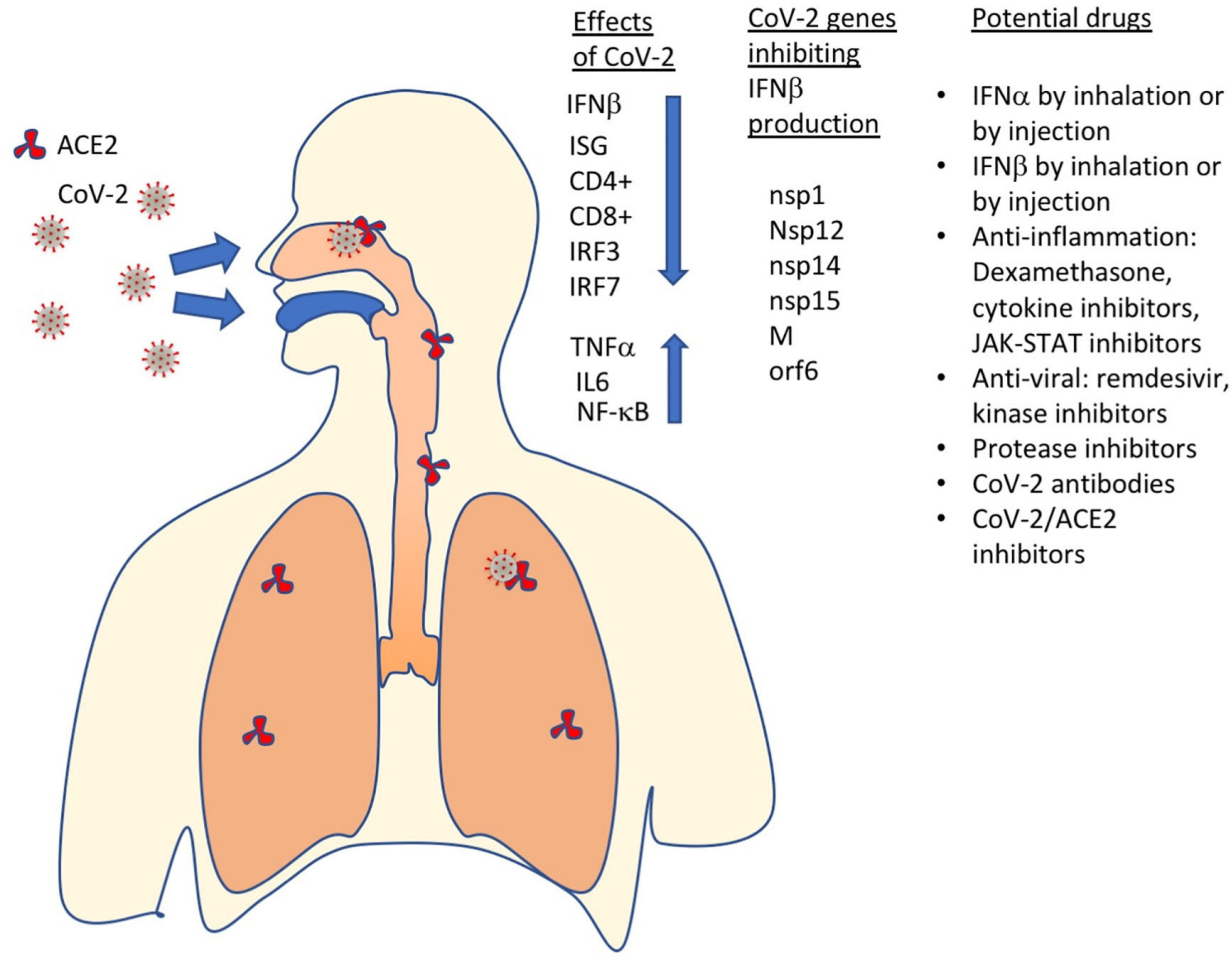
SARS-CoV-2 has multiple effects on the immune system, including inhibition of IFNβ production, which results in ISGs not to be produced, CD4+ and CD8+ exhaustion and increased levels of pro-inflammatory proteins (TNFα, IL6, NF-kB). Currently, the most promising drugs against COVID-19 include IFN-Is, anti-inflammatory and antiviral drugs, protease inhibitors, antibodies, SARS-CoV2 – ACE2 (receptor) binding inhibitors and more.

CoV-2 is presumed to infect people mostly though inhalation of viral particles, which can be airborne, in droplets or otherwise through infection through touching infected surfaces. The Spike protein on the CoV-2 surface binds to the human ACE2 protein, which serves as its receptor (**Figure 4**). The homotrimeric spike glycoprotein is made from S1 and S2 subunits. Its binding and subsequent cleavage by the host protease TMPRSS2 results in the fusion between cell and viral membranes and cell entry (77). Blocking the ACE2 receptors by specific antibodies voids viral entry (77–79). Interestingly, CoV-2 receptor-binding domain (RBD) exhibited significantly higher binding affinity to ACE2 than the SARS-CoV RBD, which was speculated to relate to the higher infectivity of COVID-19 in relation to SARS. After membrane fusion, the virus enters through the endosomal pathway and the viral RNA is released into the host cell. The viral RNA is then translated into viral polyproteins, which are cleaved into small products by viral proteases (papain-like protease [Plpro] and the main protease [Mpro]). Viral proteins and genome RNA are subsequently assembled into virions in the ER and Golgi and then transported and released out of the cell. The exact mechanism of viral self-assembly is still under intense investigation (80, 81).

Investigating ACE2 and the viral entry-associated protease TMPRSS2 expression levels in lung tissue and trachea has shown that TMPRSS2 is expressed in both tissues, while ACE2 is predominantly expressed in a transient secretory cell type (82). In addition, ACE2 and TMPRSS2 co-expressing cells were found within lung type II alveolar cells (which also release pulmonary surfactant), enterocytes, and nasal goblet secretory cells (83). Using single-cell RNA-sequencing, ACE2 and TMPRSS2 were found to be highly expressed also in the nasal goblet and ciliated cells (84). The inhaled virus likely binds to epithelial cells in the nasal cavity and starts replicating. The virus propagates and migrates down the respiratory tract along the conducting airways, and a more robust innate immune response is triggered. For about 80% of the infected patients, the disease will be mild and mostly restricted to the upper and conducting airways. Unfortunately, about 20% of the infected patients will progress to more severe disease and will develop pulmonary infiltrates and some of them will develop ARDS (85).

### Interferons and COVID-19

Like many other viruses, also SARS-CoV and SARS-CoV-2 have evolved mechanisms to reduce their exposure to IFN-I. In both viruses, mechanisms to block the production of IFNβ were identified. While the antiviral potency of IFN-Is on SARS-CoV is moderate, SARS-CoV-2 seems to be highly sensitive to IFN-I. This is evident by the significant reduction in viral replication observed following IFN-I treatment at both 24 and 48 h post-infection (86). In SARS-CoV-2–infected cells, IFN-I results in elevated STAT1 levels and ISG production (in contrast to SARS-CoV infected cells). This raises the question of why the innate immune system fails to combat SARS-CoV-2? The apparent answer to this is in the inhibition of IFNβ production by proteins of the SARS-CoV-2 virus. Within cells, RNA viruses are sensed by the innate immune system through three major classes of pattern recognition receptors (PRRs): Toll-like receptors (i.e. TLR-3, -7, -8), RIG-I-like receptors (RLRs), and NOD-like receptors (NLRs) (87). To identify the molecular mechanisms that block IFNβ production through activation of IRF3/7, several research groups transfected cells individually with all the CoV-2 viral genes and with either RIG I, MDA5, or MAVS (88, 89). Among the 27 CoV-2 proteins transfected to cells, they identified nsp14 and orf6 as competent suppressors of IFNβ. Yuen et al. also identified nsp13 and 15, while Lei et al. identified nsp1, nsp12 and the M protein as potent inhibitors of the MAVS pathway, leading to inhibition of IFNβ production (**Figure 4**). Orf6 was between the strongest suppressors of IFNβ production in both studies. Orf6 was also the only SARS-CoV-2 gene suppressing the activity of an interferon-stimulated response element (ISRE) promoter in both studies. Lei et al. also identified nsp1 and nsp14 as potent inhibitors of the induction of an ISRE promotor. In another study, Li et al. showed that the viral ORF6, ORF8, and nucleocapsid proteins were strong inhibitors of IFNβ production, and through this of the IFN-I innate immune response (90). In this study, ORF6 and ORF8 also inhibited induction of transcription an ISRE promotor driving a luciferase as reporter, following IFNβ treatment.

In addition to the above-mentioned SARS-CoV-2 genes, ORF3b was implicated by Konno et al. as being a potent antagonist towards IFN-I production (91). An interesting civet in this study is the finding that a natural variant, with a longer ORF3b reading frame increased disease severity in two patients. In light of the much higher than expected coding capacity of the SARS-CoV-2 genome, where many more proteins than genes were identified (92), we may find even more proteins and peptides being involved in eliminating the innate immune response, including through inhibition of IFN-I activities.

Another mechanism by which SARS-CoV-2 inhibit antiviral functions of the cell is thought the activity of the papain-like protease (PLpro), which is essential for viral polyprotein processing. This gene was found to preferentially cleave the ubiquitin-like modifier interferon-stimulated gene 15 (ISG15), which is an IFN-I induced gene with strong antiviral activity (93). This represents another layer of attenuation of IFN-I responses by SARS-CoV-2 and is similar to the mechanism previously identified for SARS-CoV (68).

Inhibition of IFNβ production by CoV-2 got further confirmation from measuring the levels of different cytokines in SARS-CoV-2–infected patients. An integrated immune analysis, including immune cell analysis, whole-blood transcriptomics and cytokine quantification on COVID-19 patients at 8 to 12 days after disease onset has shown an impaired IFN-I response that is a result of low IFN-I levels (94). This, in turn results in the low production of interferon-stimulated genes. Conversely, high levels of IL6 and TNFα were measured (**Figure 4**) (95, 96). This is in contrast to what is seen in patients infected with highly pathogenic influenza viruses. The high production of pro-inflammatory cytokines and low production of IFN-Is during SARS-CoV-2 infection suggests effective activation of NF-κB but not IRF3 and IRF7 (95). Impaired IFN-I production during severe COVID-19 may also lead to an imbalance in the pro-inflammatory versus pro-repair functions of airway macrophages. This was indeed seen in severely ill patients with COVID-19.

Other innate immune cells such as natural killer (NK) cells are also regulated by IFN-Is during coronavirus infection. Severe COVID-19 is associated with exhaustion of CD4+ and CD8+ T cells (97), which may be a result of deficient IFN-I production, as IFN-Is promote survival of T cells. An important issue to consider is that early production of IFN-Is promote efficient T cell responses, while a delayed response may inhibit T cell proliferation or their exit from lymphoid organs and thus cause their functional exhaustion. Indeed, T_reg_ cell counts in COVID-19 patients inversely correlate with disease severity (98, 99). Interestingly, transcriptomic analysis of blood, lung, and airways of CoV-2–infected patients showed that while IFNβ was indeed not highly expressed in either, a number of IFNαs were highly upregulated in the lung and airways but not in blood (100). Moreover, a clear IFN-I–induced gene expression profile was also detected for lung and airways, but not for blood (PBMCs). A similar finding of elevated IFNα but not IFNβ, during COVID-19 infection was also found by Wei et al. (101). In this study, the elevated IFN-I response was restricted to the stage in the disease were patients were in intensive care. In another study of 26 patients, of whom 5 did not produce IFN-I, those patients had higher viral load, required more aggressive medical intervention and their time of stay in the intensive care unit was longer that IFN-I producing patients (102).

PDCs are the most rapid and abundant IFN-I producers. PDCs express TLR7 and TLR9 which are important in sensing viruses. The response of PDCs to viruses, particularly IFN-I production, is significantly impaired with ageing while secretion of all other pro-inflammatory cytokines was comparable to that of younger individuals (103). This may relate to the master regulator for IFN-I production, IRF7, which expression, phosphorylation and nuclear translocation decreases with age. In addition, local neutrophil-mediated inflammation is increased with age, while cytotoxicity of NK cells induced by type I IFN-Is decreases in aged mice (104). In addition to age, other factors were also associated with reduced interferon responses. One of them is obesity, which is related to impaired IFNα and IFNβ responses, which may relate to inadequate response of obese people against viral infections (105).

### Treating COVID-19 Patients With IFN-I

Clinical trials of using IFN-I for treating corona viruses has a long history. Already in 1983, intranasal human IFNα2 was given both before and after corona virus challenge, a strain that is causing common cold. The incidence of colds, the severity of symptoms and signs, and virus replication were all reduced in subjects receiving interferon as compared with those given placebo (106). For SARS-CoV, no randomized placebo-controlled trials have been performed to test the efficacy of IFN-Is, however, comparing the clinical outcome of patients treated with IFN-α (infacon-1) with patients at different locations (not a control group) that were not treated, has suggested clinical benefits (107). These studies have raised the hope that IFN-I may be a potent drug also against COVID-19. This hope was further exuberated by the observation that externally administrated IFN-I induced a strong antiviral response, much more than that observed for SARS-CoV (86). While some of the SARS-CoV-2 proteins may affect ISG production (most notably, ORF6 and 8, see above), the main defense of SARS-CoV-2 against IFN-I innate immunity seems to be the prevention of IFNβ production, which can be substituted by external administration.

A major problem in assessing the efficiency of IFN-I against COVID-19 is the lack of a good small animal model. While such models are now under development, they are still not perfect. In a recent study, mice were infected with a replication-deficient adenovirus containing human ACE2, and then infected with SARS-CoV-2. These mice developed pneumonia, severe pulmonary pathology, and high-titer virus replication in lungs. To test the role of IFN-I in disease development, IFNAR1 KO mice were infected with SARS-CoV-2, showing higher viral titer over time. Next, the mice were treated prior to infection with Poly I:C, a strong inducer of IFN-I. This resulted in significantly diminished clinical disease and induced more rapid virus clearance (108). These results suggest that at least in a mice model, IFN-I may benefit disease recovery.

Due to the lack of a good animal model, and the availability of clinically approved IFN-I therapies, multiple clinical studies have been conducted administrating different subtypes of IFN-Is using different routes of administration (for summary see **Table 1**). In a preventive study, nasal drops of IFNα1 were given to 2,944 healthy medical staff in Shiyan City hospital, Hubei Province for 28 days to prevent SARS-CoV-2 infections. None of them developed serious side effects or was infected with CoV-2. While the study lacked a control group from the same city, overall in Hubei province 3,387 medical staff were diagnosed with COVID-19 (109). The study thus gives an indication that IFN-I may help in preventing infection for high risk medical personal.

**Table 1:** Summary of clinical trials conducted using IFN-is.

Study organizer	Aim of study	IFN subtype	Route of administration	Control group	Main findings	References
Shiyan City Hospital, Hubei, China	Preventive	IFNα1	Nasal drops	Health workers in different locations	Prevention of infection	(109)
Multi-center, Hong Kong	Hospital treatment of COVID-19 patients	IFNβ in combination with lopinavir, ritonavir, ribavirin	Subcutaneous injection	Patients not given IFNβ	Reduction in clinical symptoms	(110)
Wuhan, China	Hospital treatment of COVID-19 patients	IFNα2b in combination with arbidol	Nebulization to the lungs	Patients not given IFNα2b	Reduction in clinical symptoms	(111)
Imam Khomeini Hospital, Teheran, Iran	Hospital treatment of COVID-19 patients	IFNβ1a + standard care	Subcutaneous injection	Randomized clinical trial	No difference in clinical response, but lower mortality	(112)
Multi-center, Hubei, China	Hospital treatment of COVID-19 patients	IFNα2 + standard care	Inhalation	Retrospective study, historical control group	Early treatment reduced, while late treatment increased mortality	(113)
Synairgen, UK	Hospital treatment of COVID-19 patients	IFNβ	Inhalation	Controlled study	79% reduction in developing severe disease	ClinicalTrials.gov Identifier: NCT04385095

To test the benefit of subcutaneous injection of IFNβ on early stage patients, an open clinical trial was conducted with 127 patients, 86 were assigned to the combination of lopinavir, ritonavir, ribavirin, and three doses of 8 million international units of IFNβ, while the control group of 41 patients were given all the above except IFNβ. The median number of days from symptom onset to start of study treatment was 5 days. Patients given also IFNβ had a significantly shorter median time from the start of treatment to negative nasopharyngeal swab (5–11 days) in comparison to the control group (8–15 days). Moreover, IFNβ reduced viral load and number of significantly ill patients relative to the control group, this without significant side-effects (110).

In a medical study on the effects of treatment with IFNα2b in a cohort of confirmed COVID-19 patients, some of the 77 participants were given nebulized IFNα2b with or without arbidol while others were given only arbidol. Treatment with IFNα2b with or without arbidol reduced the duration of detectable virus in the upper respiratory tract and reduced duration of elevated blood levels of IL6 and c-reactive protein, which are inflammatory markers (111). While the study did not include a standard care group, and all patients recovered, it still provides an indication of IFN-I efficiency.

The efficiency of IFNβ1a subcutaneously injected three times weekly for 2 weeks for treatment of severe COVID-19 was tested in a randomized clinical trial. All the patients (including the control group) received standard of care, including a range of other medicines (hydroxychloroquine, antibiotics, antiviral medicine and more). While the clinical response was not significantly different between the IFNβ1 and the control groups, the 28-day overall mortality was significantly lower (19% vs. 44%) in the IFNβ1 treated group (112).

In a retrospective study of patients receiving IFNα2 through inhalation, alone or in combination with other drugs at a relative early versus late stage of the infection, it was found that those receiving IFNα2 at an early stage had a significantly lower rate of mortality. In contrast, late interferon therapy increased mortality and delayed recovery (113). The study suggests a relation between the time of IFN-I treatment and its efficiency.

Synairgen, a UK-based company, performed a controlled clinical trial of inhaled IFNβ on 221 patients and reported that compared with placebo the odds of developing severe disease during the treatment period decreased by 79% for hospitalized patients receiving SNG001, and that patients who received SNG001 were more than twice as likely to recover from the virus during the treatment period versus those randomized to placebo. These are between the best results achieved so far in curing COVID-19.

More clinical trials are now under way to evaluate IFN-I efficiency, but clearly the initial trials have been encouraging. Moreover, due to the many years of experience in treating patients with IFN-Is, the availability of the drug and its relatively modest cost make it an excellent candidate for mass treatment, once approved. However, critical questions remain concerning the use of IFN-Is for COVID-19 and other diseases (**Figure 4**). These questions relate to the optimal IFN-I subtype, drug-concentration, duration of treatment, mode of treatment and at which frequency should it be given. Ample experience exists with subcutaneously administration, which is almost the only route IFN-Is were used in the clinic. Here, non-modified IFN-Is are usually administrated two to three times weekly, while PEGylated IFN-Is are administrated once per week or less. Injection of IFN-Is will result in a systemic response, where IFN-Is were shown to have antiviral functions as well as pro and anti-inflammatory functions. Contrary, if given by inhalation, it will directly target the epithelial, and thus replace the IFNβ, which production is inhibited by the virus. Administration as nasal drops of IFNα may be an excellent prophylactic method for people at high risk. Ideally, these questions could be answered using animal models. The problem is that the disease in those is not equivalent to that observed in humans. Due to the severity of the disease and the high proven safety of IFN-Is, more clinical trials on humans, testing the many open questions related to its best mode of administration may be the fastest way forwards.

The subtype to use is another important question. For multiple-sclerosis, IFNβ has been used for many years (114), as it seems to provide a better anti-inflammatory response than IFNαs. This may relate to its higher binding affinity to the interferon receptors, as has been demonstrated using a tight binding IFNα mutant (YNS-α8 tail), which binding affinity even surpasses that of IFNβ [see above (57)]. For combating viral disease, most notable hepatitis C, IFNα2 has been most commonly used (115), which was later replaced by PEGylated (long plasma half-life) IFNα2 (116). Also, for cancers IFNαs were mostly used (117). A good clinical explanation of why specific IFN-I subtypes were used is often missing, and decisions of which interferon to use may often relate to availability rather than to efficacy. Moreover, due to the specie specificity of IFN-Is, one cannot deduce from mouse experiments, which IFN-I to use in humans, as the data are not transferable (57, 118). The main difference between IFNαs and IFNβ is that the later has a stronger potency to induce antiproliferative and immunomodulatory responses (tunable), while IFNα will provide a cleaner antiviral response (robust) without the additional responses associated with IFNβ. The open question is which is desired for COVID-19 treatment, where complications arise from the exuberated immune response.

Another, important parameter is the time of intervention by IFN-I, in early or late-stage COVID-19 disease. In a recent study in mice it has been shown that prolonged IFN-I and III signaling interferes with lung repair during influenza recovery, probably through p53 induction, which reduces epithelial proliferation and healing, while early treatment protects mice (119). In SARS-CoV-2 this is further complicated by the “cytokine-storm” symptoms of severe COVID-19, as indicated by elevated IL6 and TNF-alpha levels. Whether IFN-administration, particularly IFNβ suppresses or exacerbate the SARS-CoV-2 cytokine storm needs to be urgently determined, as to provide a guide for future application of IFN-I therapy in SARS-COV-2 treatment.

## Author Contributions

The author confirms being the sole contributor of this work and has approved it for publication.

## Funding

This research was supported by the Israel Science Foundation (grant No. 3814/19) within the KillCorona–Curbing Coronavirus Research Program and by the Ben B. and Joyce E. Eisenberg Foundation.

## Conflict of Interest

The author declares that the research was conducted in the absence of any commercial or financial relationships that could be construed as a potential conflict of interest.
